# Juggling conflicting demands: registered nurses’ experiences of goal conflicts in acute illness consultations in primary care – a thematic analysis

**DOI:** 10.1186/s12875-026-03364-9

**Published:** 2026-05-18

**Authors:** Malin Östman, Karin Bergman, Sofia Östensson, Inger K Holmström

**Affiliations:** 1 General Practice/Family Medicine, School of Public Health and Community Medicine, Institute of Medicine, Sahlgrenska Academy, University of Gothenburg, Box 453, Gothenburg, SE-405 30 Sweden; 2 Research, Education, Development & Innovation, Primary Care, Region Västra Götaland, Vänersborg, Sweden; 3 Department of Health Sciences, Innovation and Design, Mälardalen University, Västerås, Sweden; 4 Faculty of Caring Science, Work Life and Social Welfare, University of Borås, Borås, Sweden; 5 Department of Public Health and Caring Sciences, Uppsala University, Uppsala, Sweden

**Keywords:** Clinical assessment, Decision-making, Focus group discussions, Goal conflicts, Primary care, Registered nurse, Thematic analysis, Triage

## Abstract

**Background:**

Primary care includes preventive and acute care, and management of long-term conditions. In Sweden, approximately 40 million annual visits are made to primary care, of which more than 12 million are registered nurse consultations. Registered nurses are often responsible for initial assessments, including triage and prioritization. Timely and accurate assessments are essential, as inappropriate decisions may adversely affect both care quality and patient outcomes. However, demographic changes, rising chronic disease prevalence, and growing expectations for accessible and efficient services continue to strain primary care capacity. Therefore, this study aims to explore registered nurses’ experiences of goal conflicts in decision-making during primary care consultations for acute illnesses, with a focus on clinical assessment, prioritization, and their perceived consequences.

**Methods:**

A qualitative study using semi-structured focus group discussions was conducted to explore registered nurses’ experiences and perspectives. Seven focus group discussions with 27 registered nurses in Sweden were conducted between April and May 2025. A thematic analysis was conducted.

**Results:**

The results present one overarching theme, *Juggling conflicting demands of optimal and timely care with limited resources*, and four interrelated themes illustrating these goal conflicts: *organizational demands versus quality of care*,* accessibility versus risk of crowding out*,* expectations versus adequate resources*,* and teamwork versus professional autonomy.* The findings highlight conflicting organizational, professional, and patient-driven goals that result in ethical dilemmas and ambivalence among registered nurses.

**Conclusions:**

This study indicates that goal conflicts in primary care arise from extensive assessment responsibilities placed on registered nurses without adequate alignment with education, competence, and organizational support. Despite extensive responsibilities for clinical assessment, decision-making, and prioritization, registered nurses have limited influence over organizational conditions, creating an asymmetry between responsibility and authority. These findings suggest a need for improved alignment between roles, competence, and organizational support.

**Supplementary Information:**

The online version contains supplementary material available at 10.1186/s12875-026-03364-9.

## Background

 Primary care (PC) is the cornerstone of the healthcare system, providing accessible, continuous, and person-centered services across a broad range of conditions [1]. In Sweden, PC encompasses preventive, acute, and long-term care and accounts for approximately 40 million annual visits, over 12 million of which are to registered nurses (RNs) [2].

The scope of practice of RNs is guided by professional and ethical frameworks that emphasize autonomy, accountability, and decision-making in complex clinical contexts [3]. In Swedish PC, RNs are systematically integrated as frontline providers and frequently constitute the first point of contact for patients seeking care, alongside general practitioners (GPs) and other professionals [4]. These RNs hold general or specialist qualifications, most commonly in public health nursing [5]. They conduct clinical assessments, including triage and prioritization, and increasingly undertake autonomous consultations. This reflects an expanded scope of practice, partly driven by workforce challenges, including GP shortages and task-shifting within PC teams [6, 7].

Previous research shows that RN-led care contributes to improved quality of care and patient outcomes in PC [6, 8]. However, this role expansion has occurred without corresponding formalization of competencies or educational frameworks, raising questions regarding role clarity and scope of practice [9, 10]. While advanced nursing roles are well established in some countries, their implementation varies across Europe and remains limited in Sweden [10].

At the same time, PC faces increasing pressure from demographic changes, rising chronic disease prevalence, workforce shortages, administrative demands, and limited accessibility, with Sweden performing relatively poorly in international comparisons of PC access and continuity [11, 12]. These challenges highlight the need for efficient patient flow and optimal use of professional competence.

Within this context, triage is a key organizational process that directs patients to appropriate care levels and professionals [6, 13–15]. In Swedish PC, RNs perform not only triage but also broader clinical assessment and decision-making, including evaluating urgency, prioritizing needs, providing advice, routing patients within the system, and initiating care [13, 16, 17]. This work requires advanced clinical judgment and prioritization skills, alongside decision-support tools [18, 19].

RNs assess a wide range of health problems, including acute illnesses, most of which are managed within PC [20]. These range from potentially life-threatening conditions to self-limiting problems such as infections, gastrointestinal symptoms, and minor injuries, underscoring the breadth of RNs’ diagnostic responsibility [21, 22]. In these encounters, RNs are also expected to provide care that is responsive to patients’ individual needs and perspectives, in line with principles of person-centred care, which emphasize respect for autonomy, individual values, and shared decision-making, including patient involvement in care and management when appropriate [23]. Accurate and timely assessments are critical, as inappropriate decisions may negatively affect care quality and patient outcomes [24].

However, clinical decision-making often occurs under organizational and time constraints, creating goal conflicts, situations in which competing objectives interfere with one another and may cause stress or reduced well-being [25–27]. For RNs, these conflicts can involve balancing high-quality, ethically sound care with organizational efficiency and limited resources [28]. Ethical dilemmas in nursing, commonly described as conflicts between competing values in prioritization, are closely related to such goal conflicts [3, 28, 29]. However, research in PC appears limited and fragmented [29].

Internationally, RNs in PC have increasingly expanded roles within multiprofessional teams, contributing to coordination and continuity of care [30, 31]. However, RN-led clinical assessment and prioritization of acute illness in PC remains underexplored compared with telephone triage or chronic disease management [8, 32, 33]. In Sweden, increasing demands for accessibility and a growing number of consultations for minor health problems have intensified the demands on RNs’ assessment and prioritization work [13, 34].

As first-line providers, RNs increasingly conduct telephone, digital, and in-person consultations requiring complex clinical assessments and prioritization [18]. In this role, they must balance patient needs, organizational goals, and limited resources [35]. Despite the introduction of RN consultations to improve accessibility and efficiency, there is limited knowledge of how RNs experience and manage these competing goals in everyday clinical practice.

Therefore, this study aims to explore RNs’ experiences of goal conflicts in decision-making during PC consultations for acute illnesses, with a focus on clinical assessment, prioritization, and their perceived consequences.

## Methods

### Design

An explorative qualitative approach was adopted, using semi-structured focus group discussions (FGDs) with RNs in PC to obtain rich data on the phenomenon under study [36].

### Setting and sampling

In Sweden, PC is funded through a tax-based healthcare system that provides equal access for everyone living or working in the country. Responsibility for healthcare organization and financing is decentralized to regional and municipal levels. PC is delivered by both public and private providers within a patient choice framework, emphasizing team-based care involving GPs and RNs in collaboration with other healthcare professionals. However, challenges such as GP shortages and socioeconomic disparities remain [37].

The study was conducted at Swedish primary care centers (PCCs). To ensure a comprehensive and inclusive understanding of the phenomenon, purposive sampling was used to recruit RNs with experience in clinical assessment and prioritization within acute illness consultations. Participants were included regardless of age, sex, or nationality. In accordance with regional guidelines, access to RNs was obtained via managers at ten PCCs. Study information was distributed via email in February 2025, requesting that all eligible RNs should be informed about the study and facilitating the participation of approximately 4–6 RNs per centre in one FGD. Each PCC had between 6 and 12 RNs who met the inclusion criteria. Participation was voluntary. A reminder was sent in March, followed by telephone follow-up in April–May for those who had not responded to the emails. The invited PCCs had previously participated in the PINPOINT project [38].

Of the ten invited PCCs, two centers declined participation due to heavy workloads, and two had to withdraw their scheduled FGDs because of long-term RN sick leave. In the end, six PCCs participated, representing a mix of public and private providers in both rural and urban settings. Managers informed eligible RNs, shared study information, coordinated participation, and scheduled FGDs.

### Data collection

Seven FGDs with a total of 27 RNs were conducted between April and May 2025, with 2–7 participants in each group. Each RN participated in one FGD. At one PCC, organizational restraints resulted in two FGDs with two RNs each. All FGDs were conducted in person at each PCC, with time and location scheduled according to organizational convenience. Participant and focus group characteristics are presented in Table 1.

**Table 1:** Participant and focus group characteristics

	Number
Participating center: (public/private)	6 (5/1)
Center size: (patients)	7.000–12.000
Participating RNs:	27
Age: (years)	31–66
Sex: Female Male	25 2
Education: RN RN with specialist qualification (Public Health Nurse, Diabetes Nurse, Intensive Care Nurse, Operating Room Nurse, Midwife)	11 16
Work experiences as RN: (years)	5–44
Work experience as an RN in PC: (years)	0.5–26
Focus Group Discussions:	7
Participants (n): (FGD1 = 3, FGD2 = 6, FGD3 = 3, FGD4 = 7, FGD5 = 2, FGD6 = 2, FGD7 = 4)	2–7
Time (minutes)	47–58

FGDs were used to explore participants’ experiences through interaction, allowing participants to reflect on and build upon each other’s perspectives. This approach is suitable for examining shared clinical practices, as it can reveal both common patterns and variations, and generate deeper insights into complex clinical situations [36]. All FGDs were moderated by the first author (MÖ), who is an experienced public health nurse and researcher, with either the second author (KB) or the third author (SÖ) present as observer. In connection with the FGDs, demographic data were collected.

The interview guide was developed from existing literature and the authors’ extensive clinical experience in PC. Participants were asked to reflect on their experiences of goal conflicts in clinical assessment and prioritization of acute illness in PC. Each FGD began with an opening statement: “We are interested in your experiences of assessments, decision-making, and prioritisation regarding patients’ needs for care and treatment within acute illness”. This was followed by an open-ended question, and questions related to predefined thematic areas, supplemented with probing questions to stimulate reflection and deepen understanding of participants’ experiences (Additional file 1). This flexible approach ensured that the FGDs remained focused on the study aim while allowing exploration of issues introduced by participants and generating rich, nuanced data. Memos and field notes were used to capture key observations during interviews and to support subsequent analysis.

### Data analysis

All interviews were digitally recorded and transcribed using automated transcription software (Amberscript). The first author (MÖ) subsequently verified each transcript against the original audio recordings and made necessary corrections to ensure verbatim accuracy. Following transcription, an inductive qualitative thematic analysis was applied [39]. The process followed four iterative steps. First, the transcripts were read repeatedly with an open mind to gain familiarity with the material. Second, the text was re-read to identify meaning units that captured RNs’ experiences of challenges and goal conflicts. These units were highlighted, briefly described, and then compared for similarities and differences. Patterns were systematically grouped, and Microsoft^®^ Excel was used to facilitate organization and overview of the material. In the third step, the meaning units were condensed into descriptive texts, which were then further developed and labelled into preliminary themes. This process involved continuous writing and rewriting to refine and structure the emerging themes. Finally, the analysis was synthesized into an overall interpretation, with themes capturing RNs’ experiences of challenges and goal conflicts in clinical assessment and prioritization. Examples of the data analysis steps are presented in Table 2. Analytical transparency and data grounding were ensured by illustrating the results with verbatim quotations [39]. The first author (MÖ) conducted the initial analysis in collaboration with the last author (IKH). The emerging findings were then discussed with all authors until consensus was reached. The results were presented and further scrutinized in dialogue with other experienced researchers in the research community. Transcripts were neither returned to participants, nor were they involved in the interpretation of the findings. In addition, a reference group including members of the public and RNs from non-participating PCCs, further contributed to clarifying and refining the findings.

ChatGPT (version 5) was used to improve the readability and grammar of the text and to translate the interview guide from Swedish to English. The authors carefully reviewed, finalized, and approved all revisions. As the primary data consisted of FGDs and were analyzed using a qualitative approach, the study followed the Consolidated Criteria for Reporting Qualitative Research (COREQ) [40] (Additional file 2).

**Table 2:** Examples of meaning units, description of meaning, and themes

Meaning unit	Description of meaning	Themes
Yes, but we have a really busy schedule. It’s clear that when you feel pressured and stressed, you can miss things. // Stress definitely leads to overlooking things or not taking the time to listen properly. (FGD D)	A stressful work situation increases the risk of misjudgments.	*Organizational Demands * *versus * *Quality of Care*
Digital accessibility may lead to unintended exclusion for some, particularly the most vulnerable. (FGD A)	Availability for some pushes out others.	*Accessibility * *versus * *Risk of Crowding Out*
We sometimes end up in discussions when we refer patients to rehab or physiotherapists, but they are rarely satisfied. It’s not usually popular. // “No, I want to book a GP’s appointment. (FGD C)	Expectations of a GP’s appointment but being referred to another profession.	*Expectations * *versus * *Adequate Resources*
Yes, but there will be mutual exchange. Sometimes the GPs ask us about things too, because we’re more used to wounds and certain other issues. (FGD B)	Mutual exchange, making use of each other’s competencies.	*Teamwork * *versus * *Professional Autonomy*

## Results

**Fig. 1:**
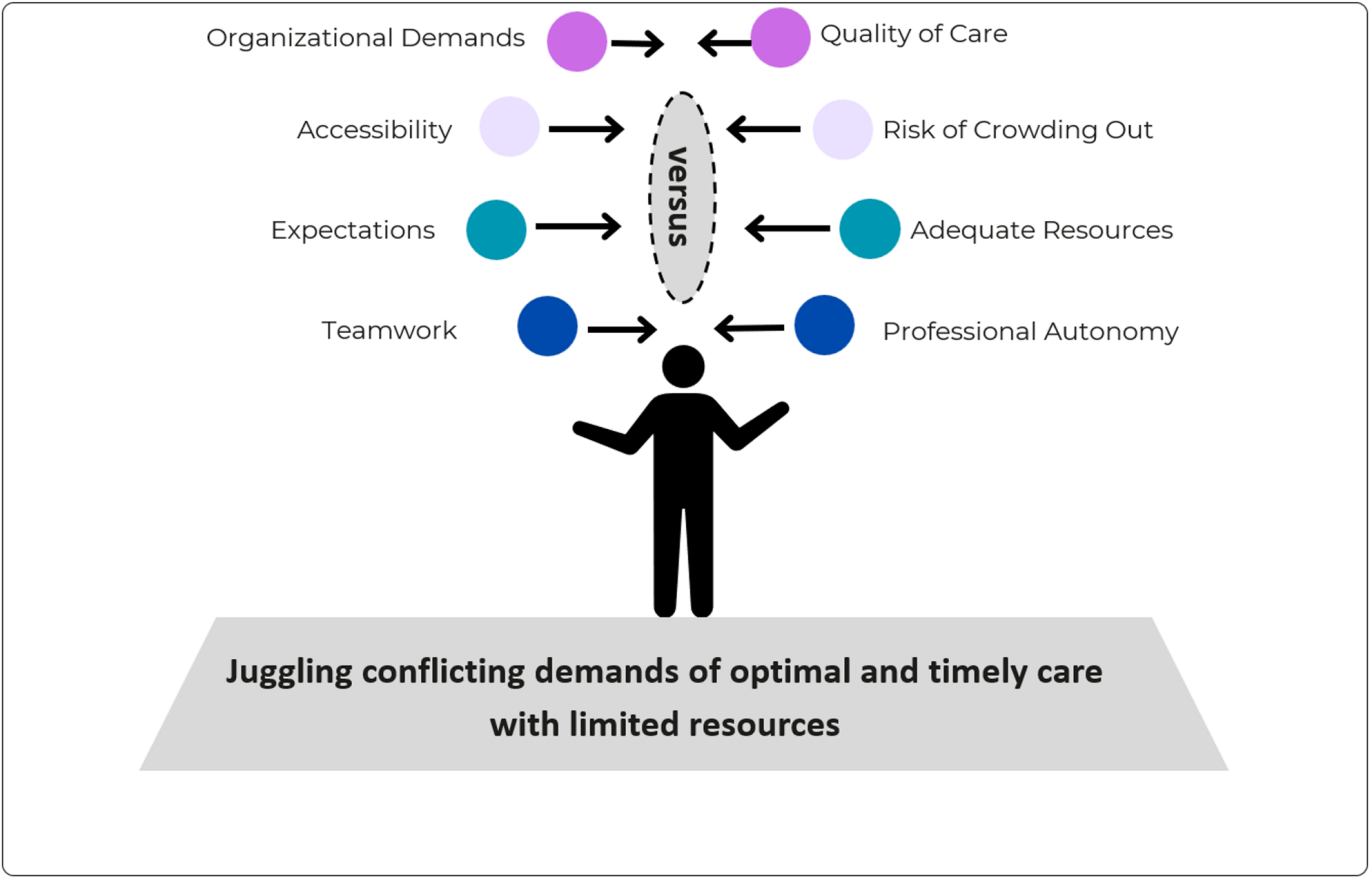
Overview of themes

While RNs in PC strive to provide accessible, equitable, and person-centered care that ensures continuity and coordination, the findings reveal inherent goal conflicts in everyday practice. The analysis identified one overarching theme, *Juggling conflicting demands of optimal and timely care with limited resources*, and four interrelated themes illustrating these goal conflicts: *organizational demands versus quality of care*,* accessibility versus risk of crowding out*,* expectations versus adequate resources*, and *teamwork versus professional autonomy.* An overview of the themes is presented in Fig. 1.

### Juggling conflicting demands of optimal and timely care with limited resources

*Juggling conflicting demands of optimal and timely care with limited resources* captures how RNs in PC routinely confront goal conflicts during consultations. The overarching theme highlights their continual navigation between professional responsibility, patient expectations, and organizational demands, often expressed as competing demands. These pressures require rapid and sometimes ethically sensitive decisions in clinical assessment and prioritization, affecting the RNs’ ability to deliver safe, person-centered, and timely care.

### Organizational demands versus quality of care

This theme describes RNs’ experiences of goal conflicts that arise when organizational demands conflict with their commitment to providing high-quality, person-centered care. Their work often requires rapid decision-making, prioritization and continuous optimization of healthcare resources, generating conflicting demands that increase stress and raise concerns about patient safety.

Organizational pressures for productivity and rapid patient flow frequently limit time for reflection and comprehensive assessment, creating tensions that lead to frustration and ethical challenges. As a result, RNs continually need to balance organizational constraints with their professional responsibility to safeguard care quality.

*That we make the right prioritizations*,* that whoever is booked is booked on the right grounds. That we schedule the patient at the right time*,* in the right place*,* and with the right professional. There is constant pressure to ensure that the right patients are at the triage unit*,* and the right patients are scheduled with GPs. But at the same time*,* we rarely receive feedback on our bookings*,* whether they have been appropriate or not. However*,* sometimes we get comments like*,* “This patient shouldn’t have come here”. (FGD E)*

Digital decision-support systems are designed to support clinical assessment and patient safety by promoting adherence to quality standards and evidence-based clinical guidelines. However, RNs often experience these systems as complex, time-consuming, and poorly aligned with clinical practice, creating conflicting demands that contribute to ethical stress and professional dissatisfaction. The systems do not adequately account for patients’ individual factors such as age, comorbidity, or social context, and their recommendations sometimes conflict with RNs’ experience-based clinical judgment. Limited access to GPs further intensifies these tensions, forcing RNs to make decisions and prioritizations that diverge from system guidance.

*I think the decision-support system is too rigid. Patients often describe far more nuance than what fits into the predefined categories. For example*,* the system states that a patient over 50 with a newly developed headache should be assessed immediately. But then you hear that they haven’t slept for a week*,* are very stressed at work*,* and may not be eating or drinking regularly. Should you book that patient then? The decision‑support system is rigid in that sense; if you were to follow it strictly*,* you would book the patient*,* but in practice that is not feasible. (FGD B)*

RNs also describe goal conflicts related to organizational expectations for digital communication. Written chat messages are time-consuming and often lack the detail needed for reliable clinical assessment, resulting in additional follow-up and delays. Without auditory or visual cues, interpreting symptoms becomes more uncertain and increases the risk of misjudgment, which in turn may undermine patient safety.

*I had a really long chat*,* and in the end*,* I had to call back because it was taking so long. Based on the assessment*,* I felt that this patient should come in. But when I spoke to the patient on the phone*,* it was something completely different*,* and I got a very different picture of the patient. You must expect that too; you even take away the voice. // Some people aren’t very good at writing*,* and sometimes they don’t answer the questions. Then you must ask again*,* which takes a lot of time. // It takes away patient safety. (FGD B)*

Finally, RNs report a goal conflict arising from increasing administrative tasks, such as billing, coding, and documentation, which consume time that would otherwise be devoted to patient care. The focus on financial and organizational demands constrains clinical practice, shifts attention away from patient needs, and leads to frustration over reduced opportunities to provide core nursing care.

*We’re really struggling with this whole documentation issue right now. I don’t think it should be my responsibility to decide who should receive an invoice. I’m a public health nurse — my role is to assess a patient’s health*,* not determine whether they should be billed. (FGD E)*

### Accessibility versus risk of crowding out

This theme illustrates a fundamental goal conflict in RNs’ work: providing rapid physical and digital access while preventing the crowding out of other patients. Prioritizing fast access may compromise fairness in care distribution, highlighting tensions between availability and needs-based, continuous, and equitable care.

Efforts to improve accessibility through telephone services, digital channels, walk-in options, and acute illness management have expanded. However, RNs noted that these initiatives may crowd out patients with complex or long-term needs, creating conflicting demands between accessibility and equality. While increased access for minor acute illness may benefit some patients, it simultaneously reduces availability for those with more complex problems, complicating prioritization and weakening continuity of care.

*Patients who need a same-day appointment are usually managed one way or another*,* especially since we opened this triage track. // But it has become even more difficult to find an appointment for someone who needs a semi-urgent appointment*,* not today*,* but soon*,* when no suitable options are available. (FGD B)*

RNs report that the growing number of contact pathways, especially digital channels, can disadvantage older or frail patients and those with limited language proficiency, thereby complicating clinical practice. Managing multiple parallel systems is time-consuming and reduces the time available for patients with greater needs, generating frustration and ethical concerns. Although these developments are intended to improve accessibility, RNs perceive that they may instead contribute to unequal access and the crowding out of more vulnerable patient groups.

*There is so much promotion of this chat service*,* presenting it as fast and efficient. It seems to be prioritized over the phone line*,* which is frustrating. After all*,* it’s not as if Anna*,* 95 years old*,* can use the fast track. (FGD B)*

RNs also face goal conflicts when walk-in patients are added to already full schedules, resulting in the crowding out of previously booked cases. This practice further complicates prioritization, as refusing care is often difficult, leading to overbooking and unintended consequences associated with increased accessibility.

*It could have waited until later that day or another day. But then it’s more difficult to tell the patient to go home again. (FGD D)*

### Expectations versus adequate resources

This theme reflects the goal conflicts RNs encounter as they balance patient expectations, professional standards, and the need for prioritization within the constraints of limited resources. Thus, RNs continually negotiate what care is needed and what can realistically be provided.

Patients often expect rapid, solution-oriented care, which can conflict with evidence-based and wise use of resources, creating emotional and moral strain. RN consultations, frequently the first and sometimes only clinical assessment, can leave patients disappointed when their expectations do not align with available resources or established care pathways.

*Some patients call with the expectation that they will get an appointment with a GP. They do not want to discuss their issue or receive any advice; they simply state*,* “I want an appointment with a GP”. They can be quite insistent about it*,* and they are not always happy about being scheduled with one of us RNs*,* who first needs to assess whether it is something we can manage. (FGD C)*

Patient expectations and perceived urgency create tensions in the assessment process, as some may amplify symptoms to secure access to a GP. This puts RNs in a difficult position, caught between responding to patient concerns and maintaining objective, evidence-based prioritization. Exaggerated symptom descriptions and external influences, such as media messaging, further complicate assessments and increase the cognitive demands associated with ensuring safe decision-making.

*But sometimes you notice that their answers change depending on the questions you ask. If I ask whether it is hard to breathe*,* they say*,* “Yes*,* it is*,* it’s probably hard to breathe.” That’s what I find difficult: you realize that they may not always be telling the truth*,* but instead adjust their answers based on our questions. At the same time*,* you can’t deny care to someone who might need it. // It is difficult. We don’t have unlimited appointment slots*,* and of course*,* we want to prioritize those who truly need to come in. (FGD B)*

RNs noted that cultural differences, gender norms, and varying understandings of illness shaped patient expectations. At times, this leads patients to question clinical judgments or request examinations that were not medically indicated. These encounters generate conflicting demands between relational care, professional judgment, and medically justified resource allocation. They require cultural sensitivity and clear communication as RNs navigate the goal conflict between meeting expectations and upholding clinical standards.

*You try to explain that this is how things work here*,* that it’s a cold*,* not dangerous*,* and that the symptoms may return. At the same time*,* you can understand their fear and worry. In many low-resource countries where antibiotics are not readily available*,* severe infections and fever can be life-threatening. This creates concern that children who develop fever and dehydration could die*,* fears that people may carry with them from their home countries. You must understand that. (FGD G)*

Finally, RNs describe striving to provide safe and compassionate care within limited resources, requiring continuous prioritization and involving goal conflicts. When organizational constraints prevented them from delivering the care they judged necessary or prioritized as most appropriated, they experienced ethical stress arising from tensions between professional ideals and everyday realities.

*It never feels good to send a patient home when they were expecting help—that’s just how it is. Even if it doesn’t have to be a conflict*,* it still doesn’t feel right. You always want the patient to feel satisfied. It’s always hard to send someone home when they feel like it was all for nothing. (FGD D)*

### Teamwork versus professional autonomy

This theme captures a central goal conflict in RNs’ work between the benefits of teamwork and the need to maintain professional autonomy. Collaboration with GPs is essential in the complex and time-pressured context of PC; however, it may also challenge RNs’ clinical judgment and professional independence.

When collaboration functions effectively, workflows are streamlined and clinical decision-making is supported. However, differences in professional roles, communication styles, and routines may hinder cooperation and influence clinical prioritization. As a result, teamwork can both enable and constrain RNs’ autonomy in everyday practice.

*Some GPs take more time*,* some prefer to see all patients themselves*,* and others don’t want to see patients at all. There are many variations. Ultimately*,* it depends on whether you want to work as part of a team or not*,* and not everyone is suited to that*,* or wants it. (FGD A)*

RNs describe tensions in teamwork arising from differing views on which health problems should be prioritized for same-day management. Decisions regarding whether non-acute illness should be handled immediately or postponed often generate collaborative challenges, especially in the context of long waiting times for GP appointments. This places RNs in a goal conflict between acting in the patient’s best interests and maintaining effective collaboration with GPs.

*I try to address what the patient actually needs help with*,* regardless of what they initially seek care for*,* and I wish GPs approached it the same way. GPs don’t always feel the need to see a patient unless the situation is urgent—really urgent. But I think that if there is time and space*,* it should be possible to address the patient’s needs. Otherwise*,* the consequence is that the patient may have to wait a very long time for follow-up care. (FGD D)*

RNs strive to make sound clinical judgments, yet limited GP availability sometimes results in patients requiring medical evaluation being scheduled with an RN instead. Consequently, RNs may be expected to assess acute medical illness beyond their formal scope of competence or comfort level, creating a goal conflict between the ideals of teamwork and the boundaries of professional autonomy. This situation contributes to stress, fear of missing a serious illness, and a sense of professional insecurity.

*It’s uncomfortable making assessments that fall outside our authority. We sometimes end up doing things we’re not fully trained or educated to do*,* and most difficult of all is discharging patients who are hesitant or unsure. (FGD D)*

Finally, RNs view frequent assessments and independent decision-making as central to their professional role, fostering both confidence and uncertainty. Limited time, incomplete information, and inconsistent access to collegial support may leave them unsure about their decisions. Such uncertainty, which may persist after the workday has ended, contributes to ongoing stress and concern.

*Sometimes I think you make so many assessments that it feels like it is the only job you do as a public health nurse at a PCC*,* making assessments. Sometimes I wonder*,* when the workday is done*,* did I do it right? // Because you only have a limited amount of time and cannot ask colleagues or GPs all the time*,* you still have to do it yourself. So sometimes I feel uncertain. (FGD B)*

## Discussion

This study explores RNs’ experiences of goal conflicts in decision-making during PC consultations for acute illnesses, with a focus on clinical assessment, prioritization, and their perceived consequences. The results show that RNs working in PC face multiple goal conflicts in their everyday clinical practice. These goal conflicts arise as they are *juggling demands of providing optimal and timely care with limited resources*, while striving to provide safe, equitable, and need-based care in accordance with PC’s overarching goals. Consistent with theoretical understandings of goal conflicts [22–24], the results demonstrate that organizational, professional, and patient-driven goals cannot always be achieved simultaneously, creating ethical dilemmas and feelings of ambivalence. It was evident that the individual RNs had to handle goal conflicts on the micro level, while the origins of the conflicts were created on meso- or macro level, such as time pressure, accessibility, and limited resources, reflecting broader system conditions influencing clinical practice [41]. This has also been described as contributing to ethical dilemmas and moral distress in nursing contexts [3, 28, 29]. These conditions somewhat constrained the possibility for high-quality assessments, nursing care, and optimal patient safety.

A central finding concerns the goal conflict between *organizational demands and quality of care*. RNs described strong pressure to optimize patient flow, make rapid decisions, and book the appropriate type of appointment, even when patients’ symptoms were complex or required more time to assess. Their experiences reflect the differing logics identified by Skirbekk et al. [42], where clinicians prioritize patient needs and ethical considerations while the organization emphasizes efficiency and resource allocation. These contrasting logics underpin the structural tensions observed in our results, as RNs felt pushed to meet efficiency targets even when they conflicted with professional judgment. The introduction of digital decision-support systems, intended to standardize assessments [18], further shaped these goal conflicts. Experienced as rigid, time-consuming, and poorly aligned with appointment availability, the systems created tensions between organizational demands for productivity and standardization [43] and RNs’ professional responsibility to conduct individualized clinical assessments and prioritize person-centred care based on medical need [23]. Furthermore, in the Swedish context, accountability rests with the individual healthcare professional for how care is performed in accordance with legislation [44], which may further intensify these tensions and contribute to ethical stress.

Being required to deviate from recommended pathways due to practical constraints generated moral stress and reinforced these goal conflicts. In line with international research, such organisational pressures, including high workload and efficiency demands, may limit RNs’ ability to act in accordance with their professional and ethical judgement, contributing to moral distress and potentially compromising both RN well-being and patient safety [28, 29, 45, 46]. Digital assessment and triage further increased the complexity of assessment and prioritization. As described by Rydell et al. [43], the shift from listening to reading digital messages may alter the RN’s clinical “toolbox”. These features of digital workflows may contribute to goal conflicts between efficiency-driven processes and the relational and interpretive dimensions of safe, person-centered care [23].

Goal conflicts also emerged around *accessibility versus risk of crowding out*, as RNs described striving to maintain high accessibility in line with PC policy goals while observing adverse effects on equity, another core value of PC [34]. Digital entry systems were reported to increase access for many patients, but risk excluding those with limited digital or linguistic abilities. As the volume of incoming requests increased without a corresponding expansion of available appointments, RNs were required to engage in more frequent need-based prioritizations to uphold principles of need and equity, thereby intensifying this goal conflict. This contradiction, where policies aimed at improving accessibility may inadvertently undermine equity and medical prioritization and person-centred care, has been described previously [47] and aligns with Swedish research on how RNs and GPs navigate resource constraints in PC [35].

A further goal conflict concerned conflicting demands between patient *expectations and adequate resources*, requiring ongoing prioritization of care Some patients were perceived as exaggerating symptoms to obtain GP appointments, creating uncertainty about actual medical needs and appropriate prioritization. When RNs denied appointments or recommended self-care based on medical need and resource constraints, they assumed a gatekeeping role that was experienced as emotionally and ethically challenging. This illustrates a goal conflict between responding to patient expectations and making professional judgments about prioritization, consistent with earlier research [26, 27], and reflects how interactions with patients and their relatives may give rise to ethical tensions in RNs clinical practice [28, 29]. At the same time, RNs emphasized that providing care at the appropriate level, even when misaligned with patient expectations, was understood as good service, echoing findings from telephone triage research on dual roles as carers and gatekeepers [48, 49]. This highlights the complexity of assessing patient-reported symptoms, where RNs must balance trust in patient narratives with professional responsibility for safe and fair prioritization.

The present findings indicate that goal conflicts may arise at the intersection *of teamwork versus professional autonomy*. While interprofessional collaboration is essential for complex assessments, differing clinical interpretations and organizational priorities create tensions between GPs and RNs [50]. In Swedish PC, these tensions may reflect a task-shifting process driven by increasing demand and limited GP availability, whereby responsibility for first-contact assessment and prioritization has largely shifted to RNs, primarily as an organizational necessity rather than a professional preference [4]. In parallel, RNs were expected to exercise considerable autonomy, often conducting clinical assessment and prioritization without adequate education, training, or support from colleagues or a GP. Such conditions may contribute to ethical challenges in RNs clinical practice, as insufficient preparedness or competence has been described as a contributing factor [29]. This pattern is consistent with previous triage research [18]; however, our findings extend beyond triage to encompass broader aspects of clinical assessment and decision-making. While this autonomy may reinforce professional identity, it also places RNs in challenging positions when complex patient cases require decisions beyond their formal competence or authority. More broadly, these findings suggest that responsibility for managing system-level goal conflicts is increasingly shifted onto individual RNs, who are implicitly expected to compensate for organizational shortcomings by acting beyond their designated scope, reflecting how system-level constraints are enacted and negotiated at the frontline of healthcare practice [41]. This raises questions about how the scope of practice of RNs is enacted under organizational constraints.

Taken together, these findings demonstrate how extensive assessment responsibilities placed on RNs in Swedish PC contribute to goal conflicts within the system. In Sweden, same-day assessments and prioritization of acute illness are routinely conducted by RNs regardless of whether they hold generalist or specialist qualifications. This may differ from international practice, where comparable assessment, triage, and prioritization tasks may typically be undertaken by RNs with advanced education and extended competencies [6]. Previous studies indicate that RNs with additional training in consultation, assessment, and triage are more likely to manage patients independently and with confidence than those without such education [34, 51].

The findings raise questions regarding the level of competence required for RNs performing advanced clinical assessment and prioritization in PC. Despite expectations of autonomy, the lack of formalized advanced practice roles and standardized educational frameworks may contribute to variation in competence, ambiguity in role expectations, and challenges in clinical decision-making [9, 10]. This aligns with international policy recommendations emphasizing the need to further develop advanced practice nursing roles to ensure access to high-quality care [9, 52].

The findings indicate important ethical implications, as RNs are required to make complex decisions under time and organizational constraints that may affect patient safety, equity of care, and accountability. These tensions may be understood as conflicts between core professional values, such as providing safe and high-quality care, respecting patient autonomy, and ensuring fairness, in line with the ICN framework [3]. Navigating these competing demands may also give rise to moral stress. Together, these findings highlight the need to align responsibilities with appropriate competence development, clearer role definitions, and organizational support to ensure safe and sustainable practice in PC.

### Strength and limitations

A key strength of this study was the use of FGDs, which facilitated interaction and allowed participants to build on one another’s reflections [36]. The participating RNs represented varied experiences in PC, contributing rich and nuanced data. The analysis was conducted rigorously, with continuous reflection on emerging findings, and the researchers’ preunderstandings in line with the chosen method [39]. The research team’s background as RNs with experience in clinical practice and PC research strengthened the study’s credibility, and although it may have influenced data collection, it was perceived to foster trust rather than constrain openness.

Recruitment via managers, required by regional guidelines, represents both a methodological and ethical limitation. This approach may have introduced selection bias by restricting participation to RNs accessible through managerial channels. It may also have influenced perceived voluntariness, as participants could have experienced implicit pressure. To mitigate this, participants were informed of the voluntary nature of participation and their right to withdraw.

FGDs of 4–6 participants are often described as balancing interaction and depth, although optimal group size may vary depending on context and study aims [36]. In this study, group sizes ranged from 2–7participants, reflecting variations in organizational conditions and staff availability across PCCs. Smaller groups may have enabled deeper discussions, whereas larger groups may have broadened perspectives but limited individual contributions. Overall, the number of FGDs was considered sufficient to support data adequacy in inductive qualitative research [53].

FGDs also entail ethical challenges, as confidentiality cannot be fully guaranteed and participants may influence each other’s willingness to speak openly [36]. Participants were therefore informed about respecting each other’s contributions, and efforts were made to create a permissive environment.

Member checking was not conducted. While often used to enhance credibility and participant involvement, it has been questioned as a validation strategy [54]. Instead, credibility was supported through ongoing discussions of the analysis and findings with a reference group and other researchers, enabling critical reflection and strengthening trustworthiness.

Finally, the Swedish PC context may limit transferability, as the roles and responsibilities of RNs vary across countries, although similar goal conflicts are likely to exist in other settings.

### Conclusion and clinical implications

This study indicates that goal conflicts in PC arise from extensive assessment responsibilities placed on RNs without adequate alignment with education, competence, and organizational support. Although RNs exercise extensive clinical assessment, decision-making, and patient prioritization, they have limited influence over the organizational conditions shaping these decisions, creating an asymmetry between responsibility and authority. Addressing these conflicts requires clearer alignment between RN roles, competence, and education, alongside strengthened training, structured clinical support, and ongoing development in assessment and prioritization, including more advanced levels of practice. Implementing clearer guidelines and strengthening team-based support may further assist RNs in clinical decision-making. Digital systems and organizational incentives should also be adjusted to support need-based medical prioritization. Otherwise, responsibility for managing these conflicts will continue to be placed on individual RNs, with implications for both patient safety and professional sustainability.

## Supplementary Information

Supplementary Material 1: Additional file 1. Word format. Interview guide

Supplementary Material 2: Additional file 2. PDF format. Consolidated criteria for Reporting Qualitative Research Checklist (COREQ)

## Data Availability

The datasets generated and/or analyzed during the current study are not publicly available due to the sensitive nature of the data and ethical considerations, but are available from the corresponding author on reasonable request.

## Abbreviations

FGDs: Focus Group Discussions
GP: General Practitioner
PC: Primary Care
PCCs: Primary Care Centers
RNs: Registered Nurses
