# Supplementary material for: Juggling conflicting demands: registered nurses’ experiences of goal conflicts in acute illness consultations in primary care – a thematic analysis
Source: BMC Prim Care. 2026 May 18;27:196. doi: 10.1186/s12875-026-03364-9 (PMC13188788; doi:10.1186/s12875-026-03364-9)
Supplement: Supplementary file 1 — Supplementary Material 1: Additional file 1. Word format. Interview guide [file 12875_2026_3364_MOESM1_ESM.docx]

## Additional file 1. Interview guide

| Thematic Area | Questions |
| --- | --- |
| Introductory question | - Can you describe your work at the assessment clinic and the types of patients you encounter? |
| 1. Patient flow, prioritization, and allocation of care | - Can you describe how decisions are made when scheduling patients to an RN or a GP? - What factors influence these prioritization decisions? and What is expected of you in this process? - Can you give examples of situations involving difficult choices or constraints? |
| 2. Clinical assessment, decision-making, and goal conflicts | - Can you describe the decisions you make during assessment consultations and what they lead to? - What factors influence your assessments and prioritization? - How do you handle competing demands or goal conflicts (e.g. patient needs, organisational constraints, available resources)? - Are some situations more challenging than others?  Can you give examples? |
| 3. Organizational support and resources | - Can you describe what types of support (e.g. guidelines, colleagues, organisational resources) do you use when making assessments and decisions? - Is there any support you feel is lacking? Can you give examples? |
| Concluding question | - Is there anything else that influences your work with assessments, prioritization, and decision-making that we have not discussed? |
| Probing questions | - Can you please describe? - Can you please tell us more? - Can you please give an example? |
